# Identifying the challenges of online education from the perspective of University of Medical Sciences Students in the COVID-19 pandemic: a Q-methodology-based study

**DOI:** 10.1186/s12909-022-03980-w

**Published:** 2022-12-27

**Authors:** Reza Ghanei Gheshlagh, Mehrdad Ahsan, Mojtaba Jafari, Hassan Mahmoodi

**Affiliations:** 1 Clinical Care Research Center, Research Institute for Health Development, Kurdistan University of Medical Sciences, Sanandaj, Iran; 2 Social Determinants of Health Research Center, Research Institute for Health Development, Kurdistan University of Medical Sciences, Sanandaj, Iran; 3 Department of Nursing, Bam University of Medical Sciences, Bam, Iran

**Keywords:** Challenge, Student, Online education, Q methodology

## Abstract

**Background:**

Challenges of online education among students of the University of Medical Sciences during the COVID-19 disease pandemic have often gone unrecognized. This study aimed to identify online education’s challenges from the perspective of students of the University of Medical Sciences during the COVID-19 pandemic in Iran.

**Method:**

The six-step Q method was used to systematically predict the different perspectives of 31 students at the Kurdistan University of Medical Sciences, Iran.

**Results:**

Four distinct patterns of Challenges of Online Education from the Perspective of participants in the COVID-19 Pandemic were identified. Four factors, which explained 69% of the total variance, included: 1) inadequacy for practical learning (26%) 2) inadequacy of Internet and website services (17%), 3) barriers related to educational content and interaction between teacher and student (8%), and 4) lack of motivation (18%).

**Conclusion:**

The identified challenges reflect the spheres that need to be focused on in interventions to facilitate the successful implementation of the challenges of online education from the perspective of the University of Medical Sciences Students in Iran and other developing countries.

## Background

Following the announcement of the spread of COVID-19 disease by the World Health Organization (WHO) on March 11, 2020, and the imposition of social restrictions, including social distancing and the closure of universities and schools, the face-to-face education system changed to an online education system [1]. In this regard, undergraduate medical education worldwide also faced problems [2]. Medical schools in different countries were forced to stop teaching in the classroom and remove students from their clinical workplaces [3]. Therefore, education shifted to e-learning, web-based, and Internet-based [4, 5].

Changing the face-to-face educational system to an online one requires educators’ and professors’ skills and competencies [6]. Effective online education requires three components: providing key educational content, counseling, and evaluation [7]. On the other hand, effective online learning also requires careful educational planning and designing effective learning environments [8, 9]. Baticulon et al.’s study showed that changing the face-to-face educational system to the online system during the COVID-19 pandemic caused students to face individual, organizational, technological, social, and home problems [2]. Some professors even expressed frustration with their ability to provide course content to students online [10]. Also, the results of a Q methodology study have shown that students generally hate online education compared to face-to-face education [11]. The results of various studies conducted on students have shown that the lack of sufficient personal interaction between students with each other and teachers and the confusing design and organization of educational materials have been the most critical challenges related to holding online and hybrid classes [12, 13]. With the expansion of online teaching methods in higher education, university professors face technical, educational, and interpersonal barriers and demands requiring skills they do not necessarily possess [14, 15]. A recent qualitative study on university students’ views on e-learning systems during the COVID-19 pandemic revealed barriers such as limited e-learning infrastructure, lack of comprehensive rules and protocols in e-learning, insufficient familiarity with e-learning, and access barriers [16]. A recent rapid review demonstrated that lack of planning and resources, usability problems, and limited interactivity between teachers and students were among the most critical barriers to online learning in clinical medical education during the first year of the COVID-19 pandemic [17]. Also, Octaberlina’s study showed that online learning barriers during the COVID-19 pandemic among students were unfamiliarity with e-learning, slow internet connection, and physical condition [18].

To the best of our knowledge, no study has been conducted to determine online education’s potential challenges among medical university students during the COVID-19 pandemic in Iran. Therefore, this study was conducted to investigate the perceived barriers to online education from the perspective of students of Medical Sciences Universities based on the Q method.

## Methods

This cross-sectional study was performed using the Q methodology during the following six steps using Barry and Proops method [19].

### Stage 1 and 2: defining the concourse

At this stage, a concourse space was formed with the identification of the subject or idea of the study. The presented views on the issue raised for the concourse can be formed from a review of texts and experts in this field [19].

In this study, the topic and idea for the concourse were the challenges of online education during the COVID-19 pandemic. The concourse included a collection of diverse materials related to the research topic that was discussed among students. The students (P-set) who also had contributed earlier to the development of the initial set of statements. Thirty-one students participated in semi-structured **interviews**, and we tried to identify their subjectivity about the research topic using the Q method [20].

In this study, the concourse (sample of people) included students of the University of Medical Sciences (paramedical students) who had enough information about online education during the COVID-19 pandemic.

### Step 3: screening and selection of statements (Q-sample)

During the semi-structured interviews with 31 students, 70 statements were extracted about the perceived challenges of online education. The Q items were selected very carefully so that items did not overlap, and at the same time, no perspective should be missing. Therefore, the selection process takes the most time and effort of all the steps of the Q methodology. Therefore, research team removed similar unrelated, and ambiguous statements from the Q set. Finally, 50 statements were selected.

### Stage 4: selected P-set

Students who participated in the concourse (interviews) were selected as a sample of individuals to participate in sorting in the Q study (P-set). In the present study, students were selected by purposive sampling to include students who had an educational, professional, experimental relationship or previous knowledge about the subject of study. This selection of samples made the participants with more diverse mentalities enter the study. It is recommended that in Q studies, the number of participants to sort statements should be less than the number of statements around the study subject [21]. In the present study, the number of participants who ranked the challenges of online education programs was 31 (Table 1).

**Table 1:** The Q-set statements and factor arrays in the study of challenges online education amongst students

Item	Statements	Factor 1	Factor 2	Factor 3	Factor 4
1	Postponement of question design during teaching	−4	−3	−2**	− 4
2	Implement unauthorized strictures due to the possibility of fraud	0	3	2	4
3	Useless practical skills	5	3	2	4
4	lack of attractiveness, being cold and soulless	4*	0	1	1
5	Complete or partial failure of the internet services	2	5*	2	1
6	Inefficiency of cell phone in opening some applications	1	1	3	3
7	Inconsistency in the files sent in one session	−2	−2	0	−2
8	Delay in sending content by professors	−1	−1	0	−1
9	Problem logging in to the site	−1	3**	−2	−1
10	Delete some timed content	0	2**	4**	0
11	website Failures	1	4**	−1	0
12	Professors use the content of previous semesters	1	−1**	−4**	2
13	Non-standardized tests	0*	2	5**	3
14	Upload incomplete content	−1	0	1	0
15	Do not load some formats on the site	0	1	0	1
16	Low quality teacher voice	−1*	0	3	1
17	Poor educational facilities	4	2	−1**	2
18	Lack of continuous evaluation by some professors	2	1	−1	1
19	Upload content without voice	−1	−1	2*	−1
20	Unable to save online classes	−5	−4	−1**	−5
21	Possibility of fraud	0	−3	0	−3
22	Uploading content is allowed	0	1	1	1
23	Lack of Teacher-Student interaction	3	0	4	0
24	Non-competitive atmosphere	1	−3**	0	−1
25	Lack of motivation amongst students	3**	1*	−1*	5**
26	Lack of load lesson plans and courses	−3	−3	−5	−3
27	Lack of opportunity to learn practical lessons	5	5	5	4
28	Lack of active dialogue between students	3**	0	1	−1
29	Sudden outage of online system	−1	4**	0	0
30	Irregularities in the conduct of classes	−4	−5*	0**	−4
31	Some students do not have access to the cell phone	−1	−2	−3	−3
32	Insufficient time to answer tests	3	2	4	5
33	No questions asked from uploaded content	−2	0	−2	0
34	No debugging class	2	0	1	2
35	Teachers do not respond via email	−3	−2	−5	−3
36	Lack of cooperation of colleges in holding re-examinations	1	1	−3**	1
37	Lack of suitable space to meet professor	2	−1	2	0
38	Failure to provide answers to questions after each test	4	3	0**	3
39	Failure to hold online courses	−3	−5**	−3	2**
40	Lack of information to students	−2	−1	−4**	−2
41	Lack of teaching essential materials	−2	− 2	−1	−2
42	Failure to answer students’ questions	−3	0**	−4*	−2
43	Typing English takes time on Adobe Connect	−4	− 4	1**	− 4
44	Holding online classes at the wrong time	−5	−4*	1**	−5
45	Non-content related assignments	−2	−2	−2	−1
46	Lac of use videos and pictures	1*	−1	−2	−2
47	Satisfaction of professors to the final score	2*	−1	−1	3*
48	The difficulty of preparing course references	1	2	3	0
48	Delay upload final question because of heavy uploads traffic	0*	4	3	2
50	Professor’s disregard for student comments	0*	1*	−3*	−1*

*P < .05; ** *P* < .01

### Stage 5: Q-sort

At this stage, the normal distribution table in the form of a Likert scale from − 5 to + 5 was designed offline. Tips on distributing the expressions on the normal distribution table were provided. In the first stage, the purpose of the study is the number of statements selected through the interview. In the second stage, place the statements in three columns: “I agree”, “I have no opinion,” and “I disagree. In the third stage, the statements (mandatory) are distributed in the normal Likert distribution diagram (− 5 to 5+), explaining the reason for choosing the two ends of the Likert scale from their point of view and finally entering the demographic information. Thus, in Q, the sorting process is subjective [19]. In other words, sorting items in the normal distribution allow each participant to present their internal perspective through sorting.

### Stage 6: analysis and interpretation of factors

Students’ data obtained from Q sorting were entered into PQ-Method software version 2.35. The process of analysis and interpretation was performed in three stages: (a) identification of factors, (b) conversion of factors into factor arrays (c) interpretation of factors using factor arrays.

A) Factor Identification

The extraction of factors in PQ-Method software was done through the following sequential steps: (a) principal component analysis, (b) identification of latent factors, (c) varimax rotation and evaluation of loading factors for specific values above 1.00, d) estimation of the percentage of variance explained by the identified factors and (e) differentiation of interpretable factors with at least two correlated Q types [22].

B) Convert factor to factor arrays

The correlation between each Q sort and one identified factor indicates the degree of interaction between the Q sorts and the identified factors [19, 23]. The manual flagging in PQ-Method software was applied for this study. The correlation coefficients of at least 0.364 were considered as the cut-off point (the absolute value of the factor load is greater than ($\frac{2.58}{\sqrt{N}}$). That factor load was 99% significant, respectively, and the value of N was equal to the number of Q statements (*N* = 50). Sorted for identified factors [24]. Specifications specified on a factor are used to create a factor array for that factor. The factor array represents the sorting of that factor (point of view) using z-scores. The factor array for each factor determined the degree to which each expression was in the spectrum, so a more accurate interpretation of each factor (subjectivity) was obtained according to the position of each expression. (*P*-value< 0.05 vs. 0.01) is also determined from the Z score to distinguish expressions [25].

III) Factor interpretation using factor arrays

Distinct Q expressions were identified, and factors were interpreted textually. The defining expressions for a factor were those that had a rating value of “+ 5”, “+ 4”, “4-,” 5- “in factor arrays that had different scores (*P* < 0.05) in a given factor Compared to their scores on other factors, the post-P-set interview was conducted at the end of Q sorting to confirm the diagnosis and interpretation of item subgroups among the identified factors.

## Results

The mean age of participants in this study was 22.64 (SD = 1.64). Slightly more than half (51%) of the participants were male students. The average duration of participants in distributing items in Q sorting was 21 minutes. Four factors, which explained 69% of the total variance, were extracted: 1) inadequacy for practical learning (26%) 2) inadequacy of Internet and website services (17%), 3) barriers related to educational content and interaction between teachers and students (8%), and 4) lack of motivation (18%).

The rotated matrix of factors showed that 19 students were loaded on the first factor and 12 on the second, two on the third, and 11 on the fourth. After determining the factor scores in the rotated matrix, factor arrays were calculated based on the scores to form a Q table for each factor and give a score to each of the Q options. The Q options were in order of importance and were identified for each factor (Table 1).

### Inappropriate for learning practical skills

Factor 1 accounted for 26% of the total variance and embodied prospects of 19 students. The items included in this factor were lack of opportunity to learn practical lessons [5], Useless in practical units [5], lack of attractiveness, being cold and soulless (*4), Inconsistency in the files sent in one session (**4), Failure to provide answers to questions after each test [4], Lack of active dialogue between students (**3) and Lack of teacher-student interaction [3].

### Inappropriate internet service and website

Factor 2: 12 participants loaded significantly on factor 2, which was explained as 17% of the total variance. The items consisting of this factor were the complete or partial failure of the internet services (*5), Lack of opportunity to learn practical lessons [5], Sudden outage of the online system (**4), website Failures (**4), delay upload final question because of heavy uploads traffic [4], problem logging into the site (**3) and failure to provide answers to questions after each test [3].

### Barriers to content education and interaction between teacher and students

Two students loaded on factor 3 accounted for 8% of the total variance. The items incorporated in this factor were non-standardized tests (**5), Lack of opportunity to learn practical lessons [5], delete some timed content (**4), lack of Teacher-Student interaction [4], and insufficient time to answer tests [4], low-quality teacher voice [3], and the difficulty of preparing course references (3).

### Lack of motivation

Factor 4: 11 study participants loaded significantly on factor 4, which explained 18% of the total variance. The items that consisted of this factor were lack of motivation amongst students (**5), insufficient time to answer tests [5], useless practical skills [4], and implement unauthorized strictures due to the possibility of fraud [4], the satisfaction of professors to the final score (*3), and failure to provide answers to questions after each test [3].

## Discussion

This study aimed to examine online education’s challenges and identify major ones. The first learning challenge identified was inadequate practical skills. Learning is most effective when learners participate in the learning process because the educational strategies used in the learning process lead to critical thinking and increase their level of awareness [26]. Students who take the initiative and are self-directed learn more than passive students [27]. The purpose of the training course is also important. A proper training course should involve the individual directly in the learning process. The course should include practical problem-solving activities that the learner may encounter in real life or in his future career.

The results of a study showed that the lack of uniformity of practice among professors was one of the challenges of teaching [28]. In another qualitative study that was conducted in order to explain the views and experiences of students and professors regarding the challenges of e-learning, the themes of “teachers’ unfamiliarity with e-learning systems” and “low quality of produced content” were extracted [29]. This finding is consistent with the results of our study.

On the other hand, effective hands-on learning can occur in the psychological atmosphere and learning engagement. Therefore, the results of the recent study showed that teacher and student interaction not only directly affects students’ learning effects but also influences students’ learning effects through the mediating effect of the psychological atmosphere and learning engagement [30].

The second challenge extracted in this research was inappropriate internet services and websites. The Internet has become an effective platform for providing virtual courses to fill the educational gap due to media richness, low cost, faster content updating, and interactive communication environment. Additionally, allow instructors to provide rich learning experiences for distance learners. However, this goal is sometimes impossible due to insufficient infrastructure [31–33].

A study by O’Doherty et al. (2018) showed that the lack of principled strategies and insufficient infrastructure are critical barriers to e-learning [34]. The lack of infrastructure and technology in developing countries such as Iran has become a barrier to medical education, covering many infrastructural problems, from lack of email access to intermittent Internet access [35]. In Attardi and Rogers’ study, technical issues such as poor internet connection prevented the lectures from being broadcast live [36]. The results of the studies conducted in Cameroon and Iran also pointed to infrastructural problems and shortcomings, such as problems of access to physical infrastructure and poor Internet connection [35, 37]. In a qualitative study by Tang et al. (2015), one of the themes extracted was the technical issue, which referred to infrastructural problems such as concerns about accessing websites, broken links, and the lack of support of some browsers for courses [38].

According to the present results, previous studies have shown that the main challenges of online learning during the COVID-19 pandemic were technical (internet connection and poor use of tools), methodological (content presentation), and behavioral challenges (personality) [39]. The third challenge was the barriers to teaching content and interaction between teachers and students. Results of a qualitative study by Yang et al. (2004) to investigate the students’ perception of the quality of online education led to the extraction of two themes of positive experiences (flexibility, cost-effectiveness, availability of electronic research, and easy connection to the Internet) and negative experiences (instructors’ delay in feedback, lack of Technical support from the instructor, lack of motivation, feeling of isolation, monotonous teaching methods, and poor course content [40].

The results of various studies have shown that formal or informal teacher-student interaction is associated with higher scores [41], development of cognitive and intellectual skills [42], increased perseverance [43], and academic self-concept [44]. Koo also believes that the greater the relationship between teacher and student inside and outside the classroom, the higher the student’s satisfaction [45]. The fourth challenge was a lack of motivation. Students’ interaction with themselves and with teachers is the key to the teaching-learning process that leads to participation in learning and enhances their motivation to learn [46]. Because of its multimedia, online education can develop interactions between students and themselves and professors and transform teaching-learning processes. Also, the formation of virtual classrooms and group discourse environments fosters participatory learning in students by creating opportunities for more interaction [47].

## Conclusion

In this study, inadequacy for practical learning, the inadequacy of Internet and website services, inadequacy for educational content and interaction between teacher, and student, and lack of motivation were the most important challenges of online learning during the COVID-19 pandemic. For the successful implementation of online educational programs for students of medical sciences universities, it is necessary to take measures to solve these challenges and apply the necessary interventions. Considering that in the future, with the occurrence of other pandemics, a large part of the curriculum will be offered online, it seems necessary to solve these challenges and strengthen online learning. Also, examining the challenges of online learning at the organizational and technical levels can be useful.

## Data Availability

The datasets used and/or analysed during the current study are available from the corresponding author on reasonable request.

## Abbreviation

WHO: (World Health Organization)

## References

[1] Fuchs K. The difference between emergency remote teaching and e-learning. Frontiers in Education. 2022;7. 10.3389/feduc.2022.921332.

[2] Baticulon RE, Sy JJ, Alberto NRI, Baron MBC, Mabulay REC, Rizada LGT (2021). Barriers to online learning in the time of COVID-19: a National Survey of medical students in the Philippines. Med Sci Educ.

[3] Ahmed H, Allaf M, Elghazaly H (2020). COVID-19 and medical education. Lancet Infect Dis.

[4] Moore JL, Dickson-Deane C, Galyen K (2011). E-learning, online learning, and distance learning environments: are they the same?. The Internet and Higher Education [Internet].

[5] Ruiz JG, Mintzer MJ, Leipzig RM (2006). The impact of E-learning in medical education. Acad Med.

[6] Frazer C, Sullivan DH, Weatherspoon D, Hussey L. Faculty perceptions of online teaching effectiveness and indicators of quality. Nurs Res Pract. 2017;2017. 10.1155/2017/9374189. 10.1155/2017/9374189 PMC5343272 28326195

[7] Tomei LA, DN. (2019). The impact of online teaching on faculty load – revisited: computing the ideal class size for traditional, online, and hybrid courses. Int J Online Pedagog Course Des.

[8] Gustafson KL, Branch RM, Alpert SA. Survey of instructional development models, third edition. Performance Improvement. 1998;37(5):36–9. 10.1002/pfi.4140370509.

[9] Rapanta C, Botturi L, Goodyear P, Guàrdia L, Koole M (2020). Online university teaching during and after the Covid-19 crisis: refocusing teacher presence and learning activity. Postdigital Sci Educ.

[10] Ramlo S (2021). The coronavirus and higher education: faculty viewpoints about universities moving online during a worldwide pandemic. Innov High Educ.

[11] Saini J, Brewer-Deluce D, Akhtar-Danesh N, Saraco A, Bayer I, Pitt C, et al. Using Q methodology to evaluate online anatomy education: learning in a COVID-19 context. FASEB J. 2021;35(S1). 10.1096/fasebj.2021.35.s1.05032.

[12] Abramenka V (2015). Students’ motivations and barriers to online education.

[13] Baum, Sandy and MM. The human factor: the Promise & Limits of online education. Am Acad Arts Sci MIT Press. 2019;148(4):235–254. DOI: 10.1162/daed_a_01769.

[14] Weaver D, Robbie D, Borland R (2008). The practitioner’s model: designing a professional development program for online teaching. Int J E-learning.

[15] Martin F, Kumar S (2021). Barriers in online education and strategies for overcoming them. A guide to administering distance learning.

[16] Salahshouri A, Eslami K, Boostani H, Zahiri M, Jahani S, Arjmand R (2022). The university students’ viewpoints on e-learning system during COVID-19 pandemic: the case of Iran. Heliyon..

[17] Bastos RA, dos S Carvalho DR, CFS B, Bergamasco EC, Sandars J, Cecilio-Fernandes D (2022). Solutions, enablers and barriers to online learning in clinical medical education during the first year of the COVID-19 pandemic: a rapid review. Med Teach [Internet].

[18] Octaberlina LR, Muslimin AI (2020). EFL students perspective towards online learning barriers and alternatives using Moodle/Google classroom during COVID-19 pandemic. Int J High Educ.

[19] Barry J, Proops J (1999). Seeking sustainability discourses with Q methodology. Ecol Econ.

[20] Watts S, Stenner P. Doing Q Methodology: theory, method and interpretation. Qual Res Psychol. 2005;2(1):67–91. DIO: 10.1080/13645579.2014.861957.

[21] Brown SR (1996). Q methodology and qualitative research. Qual Health Res.

[22] Watts S, Stenner P. Doing the fieldwork: participants, materials and procedure. Doing Q Methodol res theory, method interpret London, UK. SAGE Publ. 2012:69–90. 10.4135/9781446251911.n4.

[23] Bartlett JE, DeWeese B (2015). Using the Q methodology approach in human resource development research. Adv Dev Hum Resour.

[24] O’Leary K, Wobbrock JO, Riskin EA (2013). Q-methodology as a research and design tool for HCI.

[25] Newman I, Ramlo S. Using Q methodology and Q factor analysis in mixed methods research. Sage Handb Mix methods Soc Behav Res. 2010:505–30. 10.4135/9781506335193.n20.

[26] Oprea CL (2014). Interactive and creative learning of the adults. Procedia-Social Behav Sci.

[27] Moghadam Zadeh A, Aliakbari Z, Mazari E (2018). A study on the relationship between self-directed learning and organizational learning in educational organizations. Organ Cult Manag.

[28] Dargahi H, Ghasemi M (2010). Comparative study of electronic medical education in studied countries. Payavard Salamat.

[29] Jafari H, Keshmiri F, Shiri SD, Abghari K, Baghian N. Explaining the views and experiences of e-teacher and e-learners about virtual education in Yazd Shahid Sadoughi University of Medical Sciences. J Med Educ Dev. 2020. 10.18502/jmed.v15i2.4231.

[30] Sun H-L, Sun T, Sha F-Y, Gu X-Y, Hou X-R, Zhu F-Y (2022). The influence of teacher-student interaction on the effects of online learning: based on a serial mediating model. Front Psychol.

[31] Bangert AW (2008). The development and validation of the student evaluation of online teaching effectiveness. Comput Sch.

[32] Motiwalla L, Tello S (2000). Distance learning on the internet: an exploratory study. Internet High Educ.

[33] Kebritchi M, Lipschuetz A, Santiague L (2017). Issues and challenges for teaching successful online courses in higher education: a literature review. J Educ Technol Syst.

[34] O’Doherty D, Dromey M, Lougheed J, Hannigan A, Last J, McGrath D (2018). Barriers and solutions to online learning in medical education–an integrative review. BMC Med Educ.

[35] Bediang G, Stoll B, Geissbuhler A, Klohn AM, Stuckelberger A, Nko’o S (2013). Computer literacy and E-learning perception in Cameroon: the case of Yaounde Faculty of Medicine and Biomedical Sciences. BMC Med Educ.

[36] Attardi SM, Rogers KA (2015). Design and implementation of an online systemic human anatomy course with laboratory. Anat Sci Educ.

[37] Lakbala P (2016). Barriers in implementing E-learning in Hormozgan University of Medical Sciences. Global J Health Sci.

[38] Tang ACY, Wong N, Wong TKS (2015). Learning experience of Chinese nursing students in an online clinical English course: qualitative study. Nurse Educ Today.

[39] Khalil R, Mansour AE, Fadda WA, Almisnid K, Aldamegh M, Al-Nafeesah A (2020). The sudden transition to synchronized online learning during the COVID-19 pandemic in Saudi Arabia: a qualitative study exploring medical students’ perspectives. BMC Med Educ.

[40] Yang Y, Cornelius LF. Students’ perceptions towards the quality of online education: a qualitative approach. Assoc Educ Commun Technol. 2004.

[41] Kim YK. Racially different patterns of student-faculty interaction in college: a focus on levels, effects, and causal directions. J Profr. 2010;3(2).

[42] Kim YK, Sax LJ (2011). Are the effects of student–faculty interaction dependent on academic major? An examination using multilevel modeling. Res High Educ.

[43] Lau LK (2003). Institutional factors affecting student retention. Education..

[44] Cole D (2007). Do interracial interactions matter? An examination of student-faculty contact and intellectual self-concept. J Higher Educ.

[45] Kuh GD, Hu S (2001). The effects of student-faculty interaction in the 1990s. Rev High Educ.

[46] Palloff RM, Pratt K (2010). Collaborating online: learning together in community.

[47] Kharrazi ANK, Bazargan A, Sani FN, Mostafavi Z (2016). The relationship between interaction of engineering and technical Students’in E-learning environments higher education institute of Mehr Alborz and their academic performance. Majallah-i Amuzih-i Muhandisi-i Iran.

